# Modeling the Influence of Large Particles on Optical Properties of Nuclear Cataracts: Insights from Enhanced LOCS III-Based Computational Analysis

**DOI:** 10.3390/diagnostics16020286

**Published:** 2026-01-16

**Authors:** Chi-Hung Lee, Yu-Jung Chen, Yung-Chi Chuang, George C. Woo, Fen-Chi Lin, Shuan-Yu Huang

**Affiliations:** 1 Department of Electrical Engineering, Feng Chia University, Taichung 407, Taiwan; chihlee@fcu.edu.tw; 2 Ph.D. Program of Electrical and Communications Engineering, Feng Chia University, Taichung 407, Taiwan; p01676@gm.jente.edu.tw (Y.-J.C.); p02519@gm.jente.edu.tw (Y.-C.C.); 3 School of Optometry, The Hong Kong Polytechnic University and Center for Eye and Vision Research, 17W Hong Kong Science Park, Hong Kong SAR, China; george.woo@polyu.edu.hk; 4 Department of Ophthalmology, Kaohsiung Armed Forces General Hospital, 2, Zhongzheng 1st. Rd., Lingya District, Kaohsiung City 80284, Taiwan; 5 Department of Optometry, Central Taiwan University of Science and Technology, Taichung 406, Taiwan

**Keywords:** nuclear cataract, light scattering, micrometer-scale particles, LOCS III, slit-lamp simulation

## Abstract

**Background:** Nuclear cataracts cause visual degradation through light scattering by aggregated proteins and particles within the crystalline lens. Existing computational models mainly consider submicron scatterers, while the optical impact of micrometer-scale particles observed in human nuclear cataracts remains underexplored. **Objective:** This study extends a LOCS III–based computational cataract model by incorporating micrometer-scale particles and quantitatively evaluates their effects on forward and backward light scattering across nuclear cataract grades. **Methods:** A physics-based scattering model was implemented using optical simulation software (LightTools). Three particle populations—nanometer-scale (S-type), submicron-scale (M-type), and micrometer-scale (L-type)—were uniformly distributed within the lens. Retinal luminance reduction was analyzed for forward scattering, while slit-lamp-based backward scattering simulations were used to evaluate luminance distributions and chromaticity changes. Particle concentrations were varied within clinically reported ranges corresponding to LOCS III grades. **Results:** Micrometer-scale particles had minimal impact in early nuclear cataract grades but significantly increased forward scattering and luminance loss in advanced grades (NO5–NO6). Backward scattering simulations revealed pronounced luminance enhancement and yellow chromaticity shifts with increasing micrometer-scale particle concentration. One micrometer-scale particle produced a luminance-reduction effect equivalent to approximately 6–7 submicron particles, depending on cataract severity. **Conclusions:** Including micrometer-scale particles enables a more complete optical representation of nuclear cataracts, linking retinal image degradation with slit-lamp appearance. The model provides a physically grounded framework for offline analysis and reference data generation to support clinical interpretation of cataract grading.

## 1. Introduction

This research paper delves into the phenomenon of scattering particles observed in age-related nuclear cataracts. With advancing age, certain conditions prompt the degradation and aggregation of proteins and fibers within the lenses, resulting in the formation of particles. These particles scatter incident light, leading to a deterioration in the quality of the image projected onto the retina [1]. Recent experimental and computational studies have visualized and quantified forward light scatter and retinal stray-light using dedicated optical setups and simulation frameworks, further emphasizing the clinical impact of scatter-induced image degradation in ophthalmic optics [2,3].

The stray-light experienced by the retina occurs due to forward light scattering, primarily influenced by relatively large particles [4,5,6]. Moreover, the severity of nuclear cataracts is commonly assessed by comparing slit lamp images with a series of standard photographs depicting escalating opacity of the lens nucleus. These images primarily capture the backward scattered light generated by relatively small particles, which scatter light at high angles, a phenomenon readily observable in slit lamp examinations (as per Rayleigh scattering theory) [7,8,9].

Over the past decades, multiple computational approaches have been developed to model optical radiation transfer through the human crystalline lens, including particle-based Rayleigh and Mie scattering models, Monte Carlo photon transport simulations, and hybrid ray-tracing frameworks incorporating wavelength-dependent absorption, scattering coefficients, and anisotropy factors.

These models have been successfully applied to investigate retinal straylight, point-spread-function degradation, and cataract-induced visual impairment. For example, Kelly-Pérez et al. developed a computational cataract model that explicitly links particle size and concentration to retinal image degradation using scattering theory [10]. Cuadrado et al. further introduced an inhomogeneous Monte Carlo scattering model of the human lens, validated against donor-lens experimental data [11]. More recently, Méndez-Aguilar et al. incorporated Mie-theory-derived scattering phase functions into Monte Carlo simulations to investigate the optical impact of multilamellar bodies in nuclear cataracts [12]. However, most existing modeling studies primarily focus on submicron-scale scatterers and emphasize forward scattering effects, while the optical contribution of micrometer-scale particles—particularly in backward scattering relevant to slit-lamp observations and LOCS III grading—remains insufficiently explored.

Therefore, the specific problem addressed in this study is the lack of a computational framework that simultaneously accounts for both forward and backward light scattering caused by multi-scale particle populations—including micrometer-scale scatterers—within a LOCS III–based nuclear cataract model. Existing optical radiation transfer models cannot fully reconcile retinal straylight, slit-lamp appearance, and clinical opacity grading because they neglect the optical role of large scattering particles.

This study formulates and investigates this problem by integrating large-particle populations into a physics-based scattering model and quantitatively evaluating their impact on luminance and chromaticity. Anatomical and ultrastructural investigations have identified the presence of micrometer-sized multi-lamellar bodies (MLBs) within human age-related nuclear cataracts, with average radii reported around 2.7 μm and in some cases exceeding 1 μm. These large particles exhibit multilayered morphology and high refractive-index contrast, making them dominant contributors to wide-angle forward scattering and retinal straylight [13,14,15,16,17,18].

Understanding the nature of these particles is crucial for researchers investigating cataracts and associated eye ailments, as discerning particle sizes and concentrations can provide valuable insights into disease progression and potential treatment avenues. In addition, proteomic and biochemical studies have revealed that age-dependent crystallin destabilization, oxidative modifications, and protein aggregation contribute to the formation of multi-scale scattering particles, establishing an important molecular basis for modeling cataract progression [19,20,21,22].

In addition, proteomic and lipidomic analyses of aged and cataractous human lenses have shown that alterations in high-density protein–membrane fractions and glycolipid/glycosphingolipid pathways are closely associated with protein aggregation, membrane remodeling, and lens opacification, thereby providing a biochemical basis for the formation of scattering particles in age-related nuclear cataracts [23,24].

In our previous computational model of nuclear cataracts [13], our focus was on determining the concentration, size, and absorbance of particles forming nuclear cataracts at each level of the Lens Opacities Classification System III (LOCS III) [14]. However, this model predominantly addressed relatively small particles, overlooking the presence of larger micrometer-sized cataractous particles commonly observed in human age-related nuclear cataracts.

Prior modeling studies have indicated that even modest concentrations of large particles can substantially enhance forward scattering and retinal straylight, suggesting that neglecting such particles may lead to underestimation of functional visual deterioration [8,9,15,16,17].

By incorporating the existence of these larger particles into our computational framework, we aim to bridge the gap between physical light-scattering models and clinically observed nuclear cataract grading.

Through systematic analysis of varying large-particle concentrations and their impact on both forward and backward scattering, this study seeks to elucidate their contribution to nuclear opacity severity and color perception changes experienced by affected individuals. Furthermore, recent clinical imaging studies using Scheimpflug photography and swept-source optical coherence tomography (SS-OCT) have demonstrated strong correlations between nuclear density, light-scattering parameters, and LOCS III grading, suggesting that particle-level optical modeling may help explain objective density variations observed in vivo [25,26,27,28,29,30,31].

The particles in question comprise two distinct sizes: a smaller type with a 10-nanometer radius and a larger type with a radius of approximately 0.7 μm, as indicated in reference [32]. However, anatomical studies based on ocular tissues have suggested that the radius of certain larger particles may extend up to 1.35 μm [33]. These observations align with emerging evidence that nuclear cataracts contain multi-population particle systems—including small protein aggregates, intermediate vesicles, and large multilamellar structures—with each population contributing differently to forward and backward scattering [15,16,17,18]. Therefore, in this study, our aim is to integrate the existence of these larger particles into our computational model. This endeavor is crucial for unraveling the complexities of cataract formation and holds promise for improving clinical outcomes for individuals affected by this condition. Integrating a multi-scale particle model also provides a potential bridge between physical scattering behavior, clinical opacity grading, and modern AI-based cataract assessment systems that rely on slit-lamp or OCT imaging to extract scattering-related features [25,26,27,28,29].

For simplification, our approach initially evaluated the possible concentration of larger particles. After that, we proceeded to consider the impact of concentration of larger particles on determining the nuclear color and nuclear opalescence (NO-NC) grade of nuclear cataracts in LOCS III. Section 2.1 focuses on investigating the correlation between the increase in particle concentrations and the decrease in power transmittance ratio due to forward scattering. Section 2.2 analyzes luminance and chromaticity through backward scattering. It includes two topics: analyzing the impact of large-type particle concentration increases from 0% to 2% on luminance and chromaticity, and balancing luminance and chromaticity by modulating the small type (S-type), middle type (M-type), and large type (L-type) particle concentrations. In Section 3, the conclusion is drawn from the findings of the study, highlighting key insights, implications, and potential future research directions.

## 2. Methods

In evaluating the influence of larger protein particles on the optical properties of a nuclear cataract eye, all particles are assumed evenly distributed throughout the lens. In our modified computational model, there are three types of scattering particles present in the lens: a small-size type (S-type) with a 10 nm radius, a median-size type (M-type) with a radius of approximately 0.7 μm. (as defined by van den Berg [3]) and a large-size type (L-type) with a radius set 1.35 ± 0.35 μm with the exception of part particles more than 1.75 μm. According to Costello’s work [33], the larger particles are multilamellar bodies (MLBs), of which the refractive index, were assumed to be around 1.49 and 1.42 for the core and the surrounding cytoplasm, respectively. The features of real MLB are more complex, the scattering behavior of a multilayer particle with varying refractive indices for each layer significantly increases the complexity of the model, making it computationally intensive and potentially impractical for analysis. Therefore, the refractive indices of the lens material and cataract particles are simply set to 1.411 and 1.5017, respectively, in the simulation. The larger particles occupy a volume fraction of about $3\times 10^{-5}$ of the cataract according to Gilliland’s work [3]. The MLBs frequency along the optic axis ranges from 0 to 1392 per mm^3^ for aged transparent lenses and from 1392 to 16,666.7 per mm^3^ for aged cataractous lenses. We will build upon our original model to discuss, within the aforementioned concentration ranges, the impact of forward-scattered light from light-scattering particles on the brightness and imaging quality on the retina. Additionally, we will examine the extent to which backward-scattered light affects the assessment of NO-NC grades.

In this computational framework, the optical properties of the crystalline lens are described using a particle-based scattering model extended from our previously published LOCS III–based nuclear cataract model [13]. Rather than prescribing bulk absorption and scattering coefficients as independent parameters, these properties are implicitly determined by the physical characteristics of the scattering particles, including particle radius, number density, refractive index contrast, and wavelength-dependent absorption spectra. Specifically, absorption is modeled using experimentally derived, wavelength-dependent absorption spectra assigned to each cataract grade, as detailed in our previous work, and implemented through an exponential transmission formulation along the optical path. Scattering strength is governed by particle size and concentration, with spherical particles randomly and uniformly distributed throughout the lens volume, such that the effective scattering coefficient arises naturally from particle cross-section and number density. Scattering anisotropy is inherently captured by distinguishing Rayleigh-type scattering for nanometer-scale particles and non-Rayleigh (forward-biased) scattering for micrometer-scale particles, without introducing an explicit single-parameter anisotropy factor. The lens geometry is defined using a schematic eye model implemented in the optical simulation software LightTools (version 2023.03) [34], in which the crystalline lens is represented as a homogeneous volume with superimposed randomly distributed spherical scatterers. This approach is intended to capture population-level optical behavior rather than patient-specific anatomical variation, and it has been shown to reproduce clinically observed trends in retinal luminance reduction and slit-lamp appearance across LOCS III grades. All absorption spectra, particle parameters, and geometric definitions follow Ref. [13].

### 2.1. Luminance Analysis of Forward Scattering

**Figure 1:**
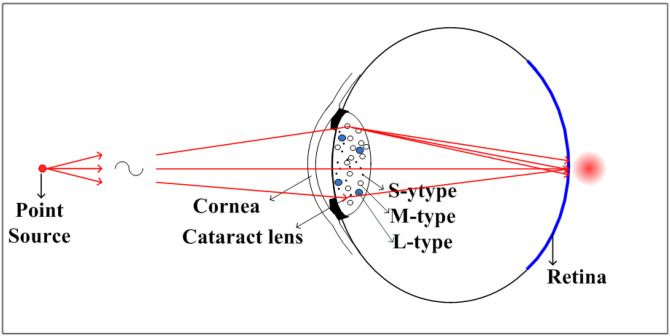
Schematic eye model with scattering particles inside the crystalline lens. The S-type, M-type and L-type particles induces scattering, primarily occurring in the forward direction, which is crucial for visual acuity.

In our original computational model (as shown in Figure 1), the concentration and the absorption spectra of particles (S-type and M-type) are specifically assigned values corresponding to NO1-NC1 through NO6-NC6 of LOCS III. As shown in Table 1, it reveals that as the particle concentration increases from <500 to 920,000 mm^−3^, the power transmittance ratio of the incident rays from a point source on a conjugate image region of the retina decreases from 1 to 0.029, which are resulted from forward scattering by the S-type and M-type particles inside the lens. In our computational model, the maximum concentration of M-type particles, occurring at 920,000 per cubic millimeter, is observed in the NO6-NC6 grade. On the other hand, the maximum concentration of L-type particles observed in a cataractous lens is 16,666.7 per cubic millimeter [2]. The ratio between these concentrations is 16,666.7/920,000 = 1.8%. Therefore, in the simulation, we set the maximum concentration of L-type particles to be 2% of the concentration of M-type particles in each grade.

In simulation, the spectral absorption coefficient of L-type particle is initially assumed equal to that of the M-type. Table 2 presents the simulated relative luminance on the retina corresponding to NO1-NC1 to NO6-NC6 of LOCS III under different combinations of S-type, M-type, and L-type particle concentrations. Item 0 lists the relative luminance from a healthy eye, which is set as a reference with a value of 1. Once the cataractous particles exist in the lens, the relative luminance is reduced due to the scattering by these cataractous particles. Items 1 to 5 display the relative luminance from a cataractous eye with a nuclear cataract grade close to NO1-NC1. As the concentration of L-type particles increases from 0% to 2% of the concentration of M-type particles, the relative luminance decreases slightly from 0.9758 to 0.9755, representing a reduction of approximately 0.03%. This suggests that the role of L-type particles is not significant and may be negligible.

Furthermore, we conducted an analysis involving five additional models: those close to NO2-NC2, NO3-NC3, NO4-NC4, NO5-NC5, and NO6-NC6. We found that as the concentration of L-type particles increased from 0% to 2% of the concentration of M-type particles, the reductions in relative luminance were 12.9%, 25.4%, 29.9%, 32.5%, and 36.1%, respectively. This suggests that as the concentration of L-type particles increases, L-type particles scatter light more effectively compared to M-type particles. This phenomenon can be attributed to the increased frequency of light bouncing back and forth in cases of higher particle concentrations.

We can quantitatively investigate the role of L-type particles from another approach: as the concentration of L-type particles increases, the concentration of M-type particles is adjusted downward to maintain the same luminance as before the introduction of L-type particles. Items 1 to 4 of Table 3 show the relative luminance on the retina corresponding to NO1-NC1 of LOCS III under different combinations of S-type, M-type, and L-type concentrations. Regardless of the specific values of M-type and L-type concentrations (denoted as M and L), the model yields the same outcome when the combinations of (M, L) are as follows: (500, 0), (400, 15), (300, 35), and (200, 50). This suggests that one L-type particle is equivalent to 6.13 M-type particles in reducing luminance. Furthermore, we conducted an analysis involving five additional models, each constructed using distinct combinations of M-type and L-type concentrations to achieve specific retinal luminance values. These values were aimed at closely resembling the cases observed in LOCS III, ranging from NO2-NC2 to NO6-NC6. In each model, the luminance reduction of one L-type particle equals a different number of M-type particles: 6.41 for NO2-NC2, 6.66 for NO3-NC3, 6.91 for NO4-NC4, 7.05 for NO5-NC5, and 7.44 for NO6-NC6, respectively, in terms of decreasing luminance. This phenomenon can also be attributed to the fact that light bounces back and forth more frequently in cases of higher particle concentrations.

### 2.2. Luminance and Chromaticity Analysis of Backward Scattering

**Figure 2:**
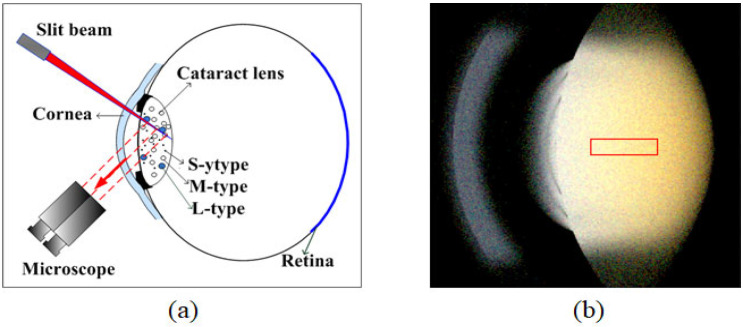
(**a**) Schematic of backward-scattering measurement using slit illumination, where the solid red arrow indicates the incident slit beam and the dashed red arrows represent backward-scattered light collected by the microscope; These captured images serve as valuable indicators for assessing the degree of cataract severity; (**b**) the ROI is indicated by a yellow rectangle.

To simulate backward scattering, the optical layout corresponding to practical slit-lamp observation is illustrated in Figure 2a. A virtual spatial luminance meter mimicked the function of a microscope by simulating the role of a receiver placed at the object plane of the microscope’s sensor. It captured the projection of a thin sheet of light onto the posterior pole of the crystalline lens, generated by a halogen slit beam set to a width of 1 mm. In analyzing the optical properties, we focus on a rectangular region along the optic axis. The analysis includes the luminance distribution and the chromaticity in the rectangle. As shown in Figure 2, the region of interest (ROI) was situated between the anterior and posterior poles, with dimensions of approximately 3.455 mm (or 33 pixels) in width and 1.047 mm (or 11 pixels) in height. After simulation by the optical software LightTools [31], the analysis proceeded through the following steps: Converting the RGB pixel values of each ROI to CIE 1931 XYZ tristimulus values. Averaging the tristimulus values vertically, maintaining a row of tristimulus values along the visual axis. Further converting the average tristimulus values into chromaticity pairs (x, y) using Equation (1), while retaining the luminance factor Y.

(1) $$\begin{array}{c} x=\frac{X}{X+Y+Z}\\ y=\frac{Y}{X+Y+Z}\\ z=\frac{Z}{X+Y+Z}=1-x-y \end{array}$$

#### 2.2.1. Analysis of L-Type Particle Concentration Increase from 0% to 2% and Its Impact on Luminance and Chromaticity

**Figure 3:**
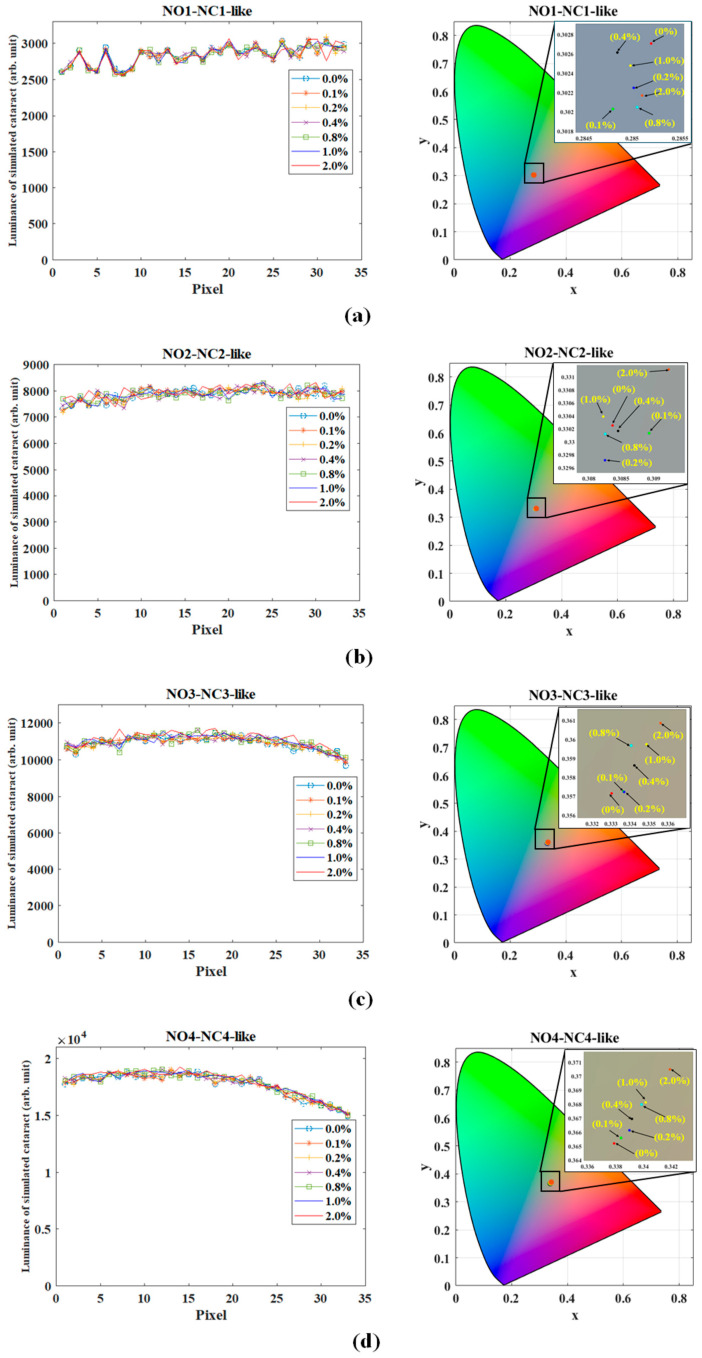

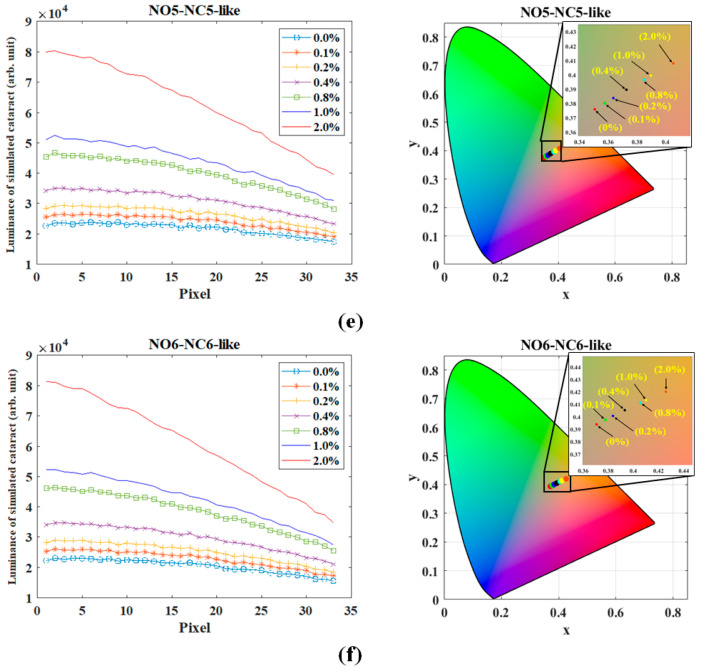
Simulated optical properties under different concentrations of L-type particles for (**a**) NO1-NC1, (**b**) NO2-NC2, (**c**) NO3-NC3, (**d**) NO4-NC4, (**e**) NO5-NC5, and (**f**) NO6-NC6 in LOCS III: (**Left**) Average luminance distribution along the visual axis, and (**Right**) corresponding centroid CIE chromaticity coordinates for cases with L-type particles ranging from 0% to 2.0%.

Figure 3a shows the luminance distribution (left picture) and the chromaticity coordinate (right picture) of NO1-NC1-like grades as the concentration of L-type particles increases from 0% to 2% of the concentration of M-type particles. The luminance distribution and the chromaticity coordinate are almost identical, suggesting that the role of L-type particles is not significant due to their low concentration. Similar results can be observed in NO2-NC2-like grades (Figure 3b), NO3-NC3-like grades (Figure 3c), and NO4-NC4-like grades (Figure 3d). As L-type particles are added to the lenses of NO5-NC5-like grades and NO6-NC6 grades, the luminance of backward scattering increases significantly. As shown in Figure 3e for NO5-NC5-like grades, with the concentration of L-type particles increasing from 0% to 2%, the average luminance increases by 192%, and the chromaticity coordinate (x, y) shifts from (0.3507, 0.3759) to (0.3897, 0.4080). Figure 3f for NO6-NC6-like grades exhibits a similar characteristic. With the concentration of L-type particles increasing from 0% to 2%, the average luminance increases by 198%, and the chromaticity coordinate (x, y) shifts from (0.3708, 0.3939) to (0.4258, 0.4201). The causes can be explained using Mie scattering theory. According to Mie theory, as the concentration of larger particles increases, multiple scattering becomes more obvious and more anisotropic, resulting in more luminance of the scattered waves in the backward direction. On the other hand, compared to shorter wavelengths (blue color), the longer wavelength in yellow (~580 nm) has more divergence in backscatter, which is beneficial for collection by the microscope, as shown in Figure 2, and induces a more pronounced yellow shift. The backward scattering shows a negative relationship with particle size and a positive correlation with wavelength.

The analysis of NO5-NC5-like grades and NO6-NC6-like grades reveals that as the concentration of L-type particles is increased from 0% to 2%, there is a marked increase in both the average luminance and the chromaticity coordinates. However, such a substantial increase in luminance is deemed uncommon in clinical settings. Consequently, it is postulated that an appropriate concentration of S-type particles would fall, wherein the luminance distribution and chromaticity coordinate remain within an acceptable range. The extreme luminance peaks observed at high S-type particle concentrations represent sensitivity boundary cases rather than typical clinical appearances, and they motivate the rebalancing analysis presented in Section 2.2.2.

#### 2.2.2. Balancing Luminance and Chromaticity Control Through Modulation of S-Type, M-Type and L-Type Particle Concentrations

**Figure 4:**
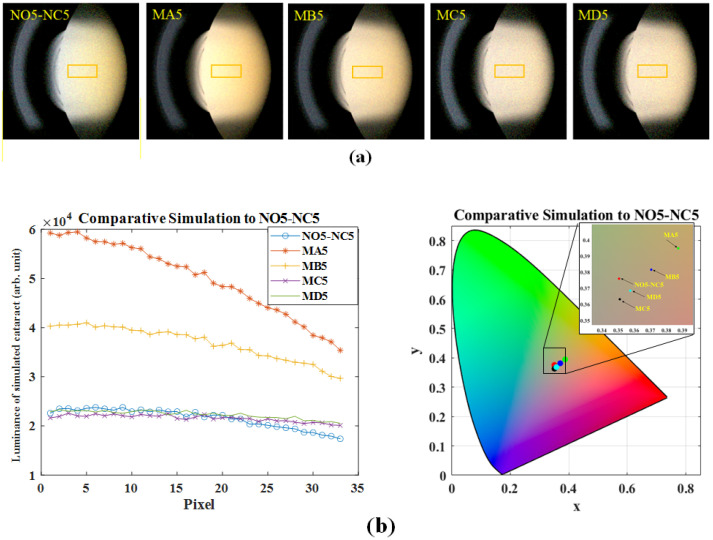
Simulated optical properties under different concentration combinations listed in Table 4: (**a**) Simulated slit-lamp photos of spatial luminance. (**b**) (**Left**) Luminance distribution along the visual axis, and (**Right**) corresponding centroid CIE chromaticity coordinates in ROI.

To find out the appropriate concentration of S-type particles, wherein the luminance distribution and chromaticity coordinates remain within an acceptable range, we ran a series of simulations for NO5-NC5 and NO6-NC6. In Table 4, we list the average luminance and chromaticity for different S-type, M-type, and L-type particle concentration combinations relative to NO5-NC5. When the concentration of L-type particles is set at 2% of that of M-type particles, decreasing the concentrations of S-type particles from 4 × 10^13^ (#/mm^3^) to 1.75 × 10^13^ (#/mm^3^) and M-type particles from 600,000 (#/mm^3^) to 225,000 (#/mm^3^) results in average luminance and chromaticity approaching those of NO5-NC5. Actually, if we further set the concentration of L-type particles at another ratio relative to that of M-type particles, we can obtain another concentration combination of S-type, M-type, and L-type particles, which also results in average luminance and chromaticity approaching those of NO5-NC5. This information suggests that a NO5-NC5 cataractous eye can be induced by different possible combinations of S-type, M-type, and L-type particles. Simulated pictures showing nuclear cataract of each combination (from case NO5-NC5 to case MD5) are shown in Figure 4a. The ROI in each photo is marked out by a yellow rectangle. The luminance distribution along the visual axis and average chromaticity in the ROI are shown in Figure 4b, revealing that though the curves of cases MC5 and MD5 have a similar average luminance, they exhibit different distribution trends along the visual axis and chromaticity coordinates due to the different interactions among these particles compared to the curve of case NO5-NC5.

**Figure 5:**
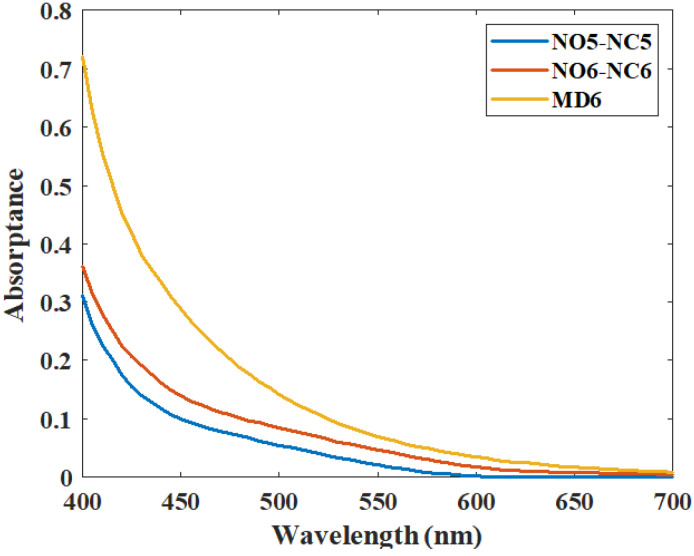
Absorption spectra for modeling cases NC5-NO5, NO6-NC6 and MD6.

**Figure 6:**
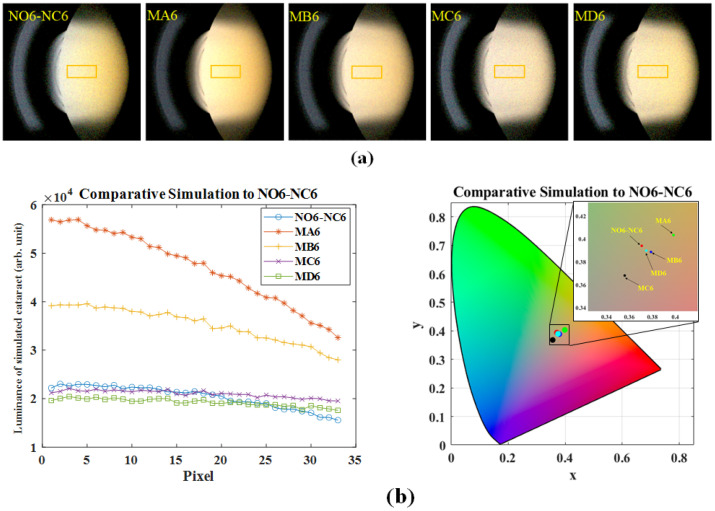
Simulated optical properties under different concentration combinations listed in Table 5: (**a**) Simulated slit-lamp photos of spatial luminance. (**b**) (**Left**) Luminance distribution along the visual axis, and (**Right**) corresponding centroid CIE chromaticity coordinates in ROI.

Table 5 lists the absorption spectra, the average luminance and chromaticity for different S-type, M-type, and L-type particle concentration combinations relative to NO6-NC6. The concentration of L-type particles is also set at 2% of that of M-type particles. When decreasing the concentrations of S-type particles from 4 × 10^13^ (#/mm^3^) to 1.75 × 10^13^ (#/mm^3^) and M-type particles from 600,000 (#/mm^3^) to 200,000 (#/mm^3^), the average luminance of cases MC6 and MD6 approaches that of NO6-NC6. However, the average chromaticity differs significantly unless we change the absorption spectra of the particles. As shown in Figure 5, the absorption spectra of the particles in case MD6 are about 2.2 times those in case NO6-NC6. This information suggests that a NO6-NC6 cataractous eye can be induced by another combination of S-type, M-type, and L-type particles but with a higher absorption spectrum. Simulated pictures showing nuclear cataract of each combination (from case NO6-NC6 to case MD6) are shown in Figure 6a. The luminance distribution along the visual axis and average chromaticity in the ROI are shown in Figure 6b.

### 2.3. Verification and Validation

To ensure numerical reliability, verification tests were conducted on the LightTools- based ray-tracing implementation. Power conservation and numerical convergence were examined by increasing the ray count until retinal luminance and chromaticity values stabilized. Sensitivity tests with respect to ray number, ROI sampling resolution, and detector binning confirmed that the predicted luminance and chromaticity trends across LOCS III grades were robust and numerically stable. The present framework is designed as a LOCS III-anchored, physics-based scattering model intended to capture grade-dependent optical behavior rather than reproduce a patient-specific experiment under identical initial conditions. Since no third-party model currently combines LOCS III particle parameterization with simultaneous prediction of forward-scatter-induced retinal luminance loss and slit-lamp-like backward-scatter luminance and chromaticity, validation was performed through consistency with established physical theory, prior computational studies, and independent clinical observations. Specifically, the simulated scattering behavior follows classical expectations: nanometer-scale particles exhibit Rayleigh-type scattering, whereas micrometer-scale particles produce strongly forward-biased, anisotropic scattering consistent with Mie theory and ocular straylight models [32,33]. The predicted role of micrometer-scale particles and multilamellar bodies (MLBs) in enhancing forward scatter and retinal straylight agrees with previously reported computational and Monte Carlo models of cataractous lenses [10,12]. Furthermore, the monotonic changes in luminance and chromaticity from NO1-NC1 to NO6-NC6 are consistent with the conceptual distinction between functional visual degradation dominated by forward scattering and slit-lamp appearance dominated by backward scattering [29,32]. Finally, the model’s grade-dependent trends are compatible with clinical imaging studies reporting strong correlations between LOCS III nuclear grades and objective lens density or scattering metrics derived from swept-source OCT and Scheimpflug imaging [8,15].

## 3. Results

All simulations were performed using the commercial optical simulation software LightTools on a standard desktop workstation (Intel i9-class CPU, 32 GB RAM). Depending on the ray number and scattering parameter settings, each simulation corresponding to a specific LOCS III grade required approximately 15 min to several tens of minutes of computation time. The computational cost scales primarily with ray count and particle concentration scenarios. Consequently, the proposed model is computationally tractable for routine offline use and is well suited for parametric analysis, mechanistic investigation of particle-induced light scattering, and generation of physically grounded reference datasets. The framework is not intended for real-time clinical deployment, but rather to support interpretation of clinical grading (e.g., LOCS III), objective imaging modalities, and emerging AI-assisted cataract assessment by providing a physics-based reference.

## 4. Discussion

The present model adopts several simplifying assumptions to ensure computational feasibility while preserving the dominant physical mechanisms governing light scattering in nuclear cataracts. First, the complex refractive index of the crystalline lens and cataractous particles is represented using effective, wavelength-dependent absorption spectra combined with real refractive indices derived from prior experimental studies. This approach avoids introducing spatially varying complex refractive index distributions, which would significantly increase model complexity without proportional gains in predictive power for LOCS III-level analysis. Second, scattering particles are assumed to be randomly and uniformly distributed throughout the lens volume. This assumption reflects a population-averaged representation of cataractous lenses and is consistent with previous computational and Monte Carlo scattering models. The present framework is therefore intended to capture grade-dependent optical trends rather than patient-specific micro-structural heterogeneity. Third, L-type particles are initially assigned absorption characteristics identical to those of M-type particles. This choice is motivated by the lack of reliable wavelength-resolved absorption data for micrometer-scale multilamellar bodies. As demonstrated in Section 2.1 and Section 2.2, forward and backward scattering effects are dominated by particle size and concentration, while absorption primarily modulates overall luminance and chromaticity. Sensitivity analyses in higher LOCS III grades further indicate that increased absorption can compensate for chromaticity deviations when large particles are present. These assumptions define the current scope of the model and highlight directions for future refinement, including incorporation of spatially inhomogeneous particle distributions, particle-specific absorption spectra, and patient-specific lens geometries when suitable experimental data become available.

## 5. Conclusions

In our original model, particle concentrations and absorption spectra were tailored to LOCS III grades. To assess L-type particle impact, simulations set L-type concentration below 2% of M-type. Forward scattering simulations reveal decreasing power transmittance ratio with rising L-type concentration, particularly noticeable in higher LOCS III grades (NO5-NC5 and NO6-NC6). Adjusting M-type concentrations to maintain consistent luminance shows one L-type particle equals at least 6.13 M-type particles in luminance reduction. In backward scattering, L-type particles have negligible effects on luminance and chromaticity in lower LOCS III grades but significantly enhance luminance in higher grades (NO5-NC5 and NO6-NC6), warranting clinical consideration. Various S-type, M-type, and L-type combinations can achieve acceptable luminance and chromaticity in cataractous eyes, offering multiple options for specific cataract grades.

## Figures and Tables

**Table 1:** Relationships Among Nuclear Cataract Type, and Simulated Particle Concentration (# denotes particle count).

Grades	S-Type Con. (#/mm^3^)	M-Type Con. (#/mm^3^)	Relative Luminance
Healthy	0	0	1
NO1-NC1	7 × 10 ^12^	5 × 10^2^	0.976
NO2-NC2	1.6 × 10^13^	2.7 × 10^5^	0.302
NO3-NC3	1.9 × 10^13^	6.1 × 10^5^	0.086
NO4-NC4	3.6 × 10^13^	7.6 × 10^5^	0.050
NO5-NC5	5 × 10^13^	8.4 × 10^5^	0.038
NO6-NC6	5 × 10^13^	9.2 × 10^5^	0.029

**Table 2:** Retinal Luminance Values for Various M-Type and L-Type Concentration Combinations (# denotes particle count).

Item #	Grades	S-Type Con. (#/mm^3^)	M-Type Con. (#/mm^3^)	L-Type Con. (#/mm^3^)	Relative Luminance
0	Healthy	0	0	0.0	1
1	NO1-NC1	7 × 10 ^12^	500	0.0	0.975805773
2	NO1-NC1-like	7 × 10^12^	500	2.5	0.975746143
3	NO1-NC1-like	7 × 10 ^12^	500	5.0	0.975651642
4	NO1-NC1-like	7 × 10^12^	500	7.5	0.975531109
5	NO1-NC1-like	7 × 10^12^	500	10.0	0.975505804
6	NO2-NC2	1.6 × 10^13^	270,000	0.0	0.301749433
7	NO2-NC2-like	1.6 × 10^13^	270,000	1350.0	0.291329185
8	NO2-NC2-like	1.6 × 10^13^	270,000	2700.0	0.281362676
9	NO2-NC2-like	1.6 × 10^13^	270,000	4050.0	0.271973768
10	NO2-NC2-like	1.6 × 10^13^	270,000	5400.0	0.262727731
11	NO3-NC3	1.9 × 10^13^	610,000	0.0	0.085914785
12	NO3-NC3-like	1.9 × 10^13^	610,000	3050.0	0.079880455
13	NO3-NC3-like	1.9 × 10^13^	610,000	6100.0	0.074117036
14	NO3-NC3-like	1.9 × 10^13^	610,000	9150.0	0.068851489
15	NO3-NC3-like	1.9 × 10^13^	610,000	12,200.0	0.064016434
16	NO4-NC4	3.6 × 10^13^	760,000	0.0	0.050030085
17	NO4-NC4-like	3.6 × 10^13^	760,000	3800.0	0.045849817
18	NO4-NC4-like	3.6 × 10^13^	760,000	7600.0	0.041714349
19	NO4-NC4-like	3.6 × 10^13^	760,000	11,400.0	0.03838217
20	NO4-NC4-like	3.6 × 10^13^	760,000	15,200.0	0.035054652
21	NO5-NC5	5 × 10^13^	840,000	0.0	0.037573552
22	NO5-NC5-like	5 × 10^13^	840,000	4200.0	0.034171572
23	NO5-NC5-like	5 × 10^13^	840,000	8400.0	0.031011324
24	NO5-NC5-like	5 × 10^13^	840,000	12,600.0	0.028103946
25	NO5-NC5-like	5 × 10^13^	840,000	16,800.0	0.025350579
26	NO6-NC6	5 × 10^13^	920,000	0.0	0.028946889
27	NO6-NC6-like	5 × 10^13^	920,000	4600.0	0.025904812
28	NO6-NC6-like	5 × 10^13^	920,000	9200.0	0.023152232
29	NO6-NC6-like	5 × 10^13^	920,000	13,800.0	0.020597675
30	NO6-NC6-like	5 × 10^13^	920,000	18,400.0	0.018486746

**Table 3:** Retinal Luminance Values for Various M-Type and L-Type Concentration Combinations to maintain similar luminance for each grade (# denotes particle count).

Item #	Grades	S-Type Con. (#/mm^3^)	M-Type Con. (#/mm^3^)	L-Type Con. (#/mm^3^)	Relative Luminance
0	Healthy	0	0	0	1
1	NO1-NC1	7 × 10^1^ ^2^	500	0	0.976
2	NO1-NC1-like	7 × 10^12^	400	15	0.976
3	NO1-NC1-like	7 × 10^12^	300	35	0.976
4	NO1-NC1-like	7 × 10^12^	200	50	0.976
5	NO2-NC2	1.6 × 10^13^	270,000	0	0.302
6	NO2-NC2-like	1.6 × 10^13^	265,000	795	0.302
7	NO2-NC2-like	1.6 × 10^13^	260,000	1560	0.302
8	NO2-NC2-like	1.6 × 10^13^	255,000	2295	0.302
9	NO3-NC3	1.9 × 10^13^	610,000	0	0.086
10	NO3-NC3-like	1.9 × 10^13^	600,000	1500	0.086
11	NO3-NC3-like	1.9 × 10^13^	590,000	2950	0.086
12	NO3-NC3-like	1.9 × 10^13^	580,000	4600	0.086
13	NO4-NC4	3.6 × 10^13^	760,000	0	0.050
14	NO4-NC4-like	3.6 × 10^13^	740,000	2960	0.050
15	NO4-NC4-like	3.6 × 10^13^	720,000	5760	0.050
16	NO4-NC4-like	3.6 × 10^13^	700,000	8540	0.050
17	NO5-NC5	5 × 10^13^	840,000	0	0.038
18	NO5-NC5-like	5 × 10^13^	820,000	2870	0.038
19	NO5-NC5-like	5 × 10^13^	800,000	5600	0.038
20	NO5-NC5-like	5 × 10^13^	790,000	7110	0.038
21	NO6-NC6	5 × 10^13^	920,000	0	0.029
22	NO6-NC6-like	5 × 10^13^	900,000	2700	0.029
23	NO6-NC6-like	5 × 10^13^	880,000	5280	0.029
24	NO6-NC6-like	5 × 10^13^	860,000	8170	0.029

**Table 4:** Comparison of Luminance and Chromaticity for Different S-Type, M-Type and L-Type Particle Concentration Combinations Relative to NO5-NC5 (# denotes particle count).

Item #	S-Type Con. (#/mm^3^)	M-Type Con. (#/mm^3^)	L-Type Con. (#/mm^3^)	Average Luminance (Arb. Unit)	Average Cx	Average Cy
NO5-NC5	5 × 10 ^13^	8.4 × 10^5^	0	21,592	0.3507	0.3759
MA5	4 × 10^13^	6 × 10^5^	12,000	49,664	0.387	0.3948
MB5	3 × 10^13^	4 × 10^5^	8000	36,880	0.371	0.3815
MC5	2 × 10^13^	2 × 10^5^	4000	21,567	0.351	0.3631
MD5	1.75 × 10^13^	2.25 × 10^5^	4500	22,295	0.358	0.3685

**Table 5:** Comparison of Luminance and Chromaticity for Different S-Type, M-Type and L-Type Particle Concentration Combinations Relative to NO6-NC6 (# denotes particle count).

Item #	Absorption Spectra	S-Type Con. (#/mm^3^)	M-Type Con. (#/mm^3^)	L-Type Con. (#/mm^3^)	Average Luminance (Arb. Unit)	Average Cx	Average Cy
NO6-NC6	Standard	5 × 10^13^	920,000	0	2.04 × 10^4^	0.3708	0.3939
MA6	Standard	4 × 10^13^	600,000	12,000	4 × 10^4^	0.3984	0.4032
MB6	Standard	3 × 10^13^	400,000	8000	3.53 × 10^4^	0.3788	0.3885
MC6	Standard	2 × 10^13^	200,000	4000	2.1 × 10^4^	0.3561	0.368
MD6	Higher	1.75 × 10^13^	200,000	4000	1.92 × 10^4^	0.3747	0.3898

## Data Availability

The data presented in this study are available from the corresponding authors upon reasonable request.
